# The Effect of SP/NK1R on the Expression and Activity of Catalase and Superoxide Dismutase in Glioblastoma Cancer Cells

**DOI:** 10.1155/2021/6620708

**Published:** 2021-04-21

**Authors:** Faranak Korfi, Hossein Javid, Reza Assaran Darban, Seyed Isaac Hashemy

**Affiliations:** ^1^Department of Biology, Faculty of Sciences, Islamic Azad University of Mashhad, Mashhad, Iran; ^2^Department of Clinical Biochemistry, Faculty of Medicine, Mashhad University of Medical Sciences, Mashhad, Iran; ^3^Department of Medical Laboratory Sciences, Varastegan Institute for Medical Sciences, Mashhad, Iran; ^4^Surgical Oncology Research Center, Mashhad University of Medical Sciences, Mashhad, Iran

## Abstract

**Introduction:**

Glioblastoma is the most malignant brain tumor with different therapeutic protocols, including surgery, radiotherapy, and chemotherapy. Substance P (SP), a peptide released by sensory nerves, increases cellular excitability by activating the neurokinin-1 receptor (NK1R) in several human tumor cells. Aprepitant is a potent and long-lasting NK1R antagonist, considered a new agent for inhibiting proliferation and induction of apoptosis in malignant cells. This study aimed to evaluate the effects of the SP/NK1R system on the expression and activity of catalase and superoxide dismutase (SOD) in the glioblastoma U87 cancer cell line.

**Methods:**

Cytotoxicity was measured by the resazurin test, 24 hours after treatment, with increasing aprepitant concentrations. The production of reactive oxygen species (ROS) was also measured 24 hours after treatment with SP and aprepitant. Enzymes activity of catalase and SOD was measured using the corresponding assay kits. Real-time PCR also measured their expression.

**Results:**

Aprepitant significantly reduced the viability of U87 cells in a concentration-dependent manner. ROS production was significantly reduced, and the activity of catalase and SOD increased after treatment with aprepitant. The expression of catalase and SOD enzymes also increased significantly in the presence of aprepitant.

**Conclusion:**

The present study showed that aprepitant inhibited SP's oxidizing effects via inducing the antioxidant effects of catalase and SOD in the U87 cell line. Therefore, this drug might be introduced as a potential candidate for controlling glioblastoma cancer in animal models and clinical trials.

## 1. Introduction

Glioblastoma multiforme (GBM) is the most common and aggressive primary brain tumor worldwide, and its occurrence is increasing [1, 2]. It is the third leading cause of death from cancer and overall in individuals aged 15–39 years [3]. In recent years, much evidence has emerged that several factors and mechanisms are involved in the initiation and progression of glioblastoma cell carcinoma, including the tachykinin family and their receptors [4, 5].

Tachykinins (TKs) include an evolutionarily conserved family of neuropeptides, widely distributed in the peripheral and central nervous systems [6]. Three receptors perform TKs G-protein coupled receptors' biological actions, named neurokinin-1 receptor (NK1R), NK2R, and NK3R [7, 8].

Substance P (SP) is the most important member of the mammalian TK peptides, and its biological effects are mainly mediated through NK1R [9, 10]. Several studies have recently demonstrated that SP induces a series of signaling pathways through NK1R that mediate cellular excitability in several human tumor cells. It has important roles in forming and spreading various tumor cells via migration, angiogenesis, and metastasis [11–16]. This is important since the prevention of metastasis is a major goal in treating tumors. Over 90% of cancer deaths are derived not only from the primary tumor but also metastasis [17, 18].

Moreover, it has recently been reported that an SP-mediated process may be the extravasation of tumor cells into the brain to form cerebral metastases [19, 20]. It is established that malignant tissues express more NK1 receptors than benign tissues and that tumor cells expressing the most malignant phenotypes display an increased percentage of NK1 receptor expression [21–26]. Moreover, the expression of SP's precursor increases in GBM cancer compared to normal cells [27]. Thus, it appears that tumor cells depend strongly on the potent mitotic signal mediated by SP and the overexpression of the NK1 receptor, leading to the death of cells [28–30]. This means the NK1 receptor might be a specific molecular target for cancer treatment since tumor cells overexpress NK1 receptors. Hence, NK1R antagonists might be considered promising therapeutic drugs to inhibit the proliferation and development of tumor cells and angiogenesis [21, 28].

On the other hand, reactive oxygen species (ROS) are an integral part of the cell oxygen metabolism, which plays a vital role in several cellular processes at physiological concentrations by activating signaling pathways necessary for cell growth and proliferation [31, 32]. Since the ROS's high levels cause destructive effects on the body, the body produces defenders that extinguish ROS shortly after their effects [33]. Collectively, these defenders or molecules are known as antioxidants. The antioxidant system consists of the enzymatic defenses, such as superoxide dismutase (SOD), which catalyzes the conversion of superoxide to H_2_O_2_ and catalase (CAT) which promotes the reduction of H_2_O_2_ to H_2_O and O_2_ [34, 35].

However, the antioxidant system's inability to regulate the ROS level results in oxidative stress involved in many diseases' pathogenesis, including cancers [36–39]. Moreover, several studies indicate crosstalk between the SP/NK1R system and the redox system. For example, Wang et al. have shown that the ROS level significantly increased in MES23.5 neuroblastoma cells after treatment with SP [40]. Consistently, Baek et al. observed that human retinal pigment epithelial (ARPE-19) cells can survive under oxidative stress by activating survival signaling pathways, including Akt. Therefore, the inhibition of Akt signaling can promote H_2_O_2_-induced cell death. In contrast, SP treatment caused Akt signaling activation; therefore, SP-activated Akt signaling might contribute to RPE cells' accelerated recovery under oxidative stress. Besides, SP's effects on the activation of signaling molecules and cell survival were mediated via NK1R [41].

Also, an in vivo study showed that the administration of an NK1R antagonist could lead to a decrease in intraabdominal adhesion by reducing the level of ROS [42].

Hence, this study aimed to explore the possible effect of the SP/NK1R system and aprepitant, a potent NK1R antagonist, on the expression and activity of catalase and superoxide dismutase enzymes, two of the most well-known antioxidant enzymes, in U87 glioblastoma cancer cells.

## 2. Materials and Methods

### 2.1. Cell Culture and Reagents

U87 glioblastoma cancer cells were purchased from Pasteur Institute, Iran. Cells were maintained in RPMI-1640 medium (Gibco, Grand Island, NY) supplemented with 10% fetal bovine serum (FBS) (Gibco, Grand Island, NY) and 1 mL of penicillin and streptomycin (Sigma 10,000 units penicillin and 10 mg of streptomycin/mL), incubated at 37°C with 5% CO_2_. SP and aprepitant were purchased from Sigma-Aldrich Company, St. Louis, MO, USA.

### 2.2. Resazurin Cell Viability Assay

The resazurin cell viability assay technique was used to evaluate the survival rate of U87 cells in the vicinity of the striatum as described before [43]. Resazurin is a weak blue fluorescence compound which reduced to a high-fluorescence, pink product (resorufin) by reducing enzymes present only in metabolically active cells. The rate of resorption of resazurin during this process is directly proportional to the number of living cells in this technique.

For this purpose, U87 cells were treated with varying concentrations (0 (control), 5, 10, 25, 35, and 50 *μ*M) of aprepitant for 24 hours. After that, the fluorescence intensity in each well was measured by a plate reader at 530 nm excitation wavelength and 590 nm emission wavelength.

### 2.3. RNA Extraction and Quantitative Real-Time PCR (qRT-PCR)

According to the manufacturer's instructions, total RNA was extracted using TRIzol reagent (Invitrogen, Carlsbad, CA) from cultured U87 cells. Complementary DNA (cDNA) was synthesized by reverse transcription with total RNA, using a reverse transcriptase cDNA synthesis kit (Takara, China). PCR products were separated by electrophoresis on agarose containing ethidium bromide. For quantitative real-time PCR (QRT-PCR) analysis, cDNA was amplified using an SYBR Green PCR Kit (Takara, China) and the Stratagene real-time PCR system (Agilent, USA). The differential expression was calculated by the 2^−ΔΔCT^ method and assessed statistically.

### 2.4. Assessment of Superoxide Dismutase (SOD) and Catalase (CAT) Activity

To measure these enzymes' activity in the U87 cell line, commercial kits from Teb Pazhouhan Razi (TPR), Tehran, Iran, were utilized. The protocol was executed following the protocol from the manufacturer. The enzyme activity was computed as enzyme/mg protein (U/mg protein).

### 2.5. Statistical Analysis

All results are presented as the mean ± standard deviation of three independent experiments. Statistical analyses were determined using ANOVA followed by Bonferroni's *t*-test for multigroup comparisons. The *p* value <0.05 was considered statistically significant for all tests. The GraphPad Prism® 6.0 software (San Diego, CA, USA) for Windows was used for all statistical analyses.

## 3. Results

The data that support the findings of this study are available from the corresponding author upon reasonable request.

### 3.1. The Effect of Aprepitant on the Survival Rate of U87 Cells

**Figure 1:**
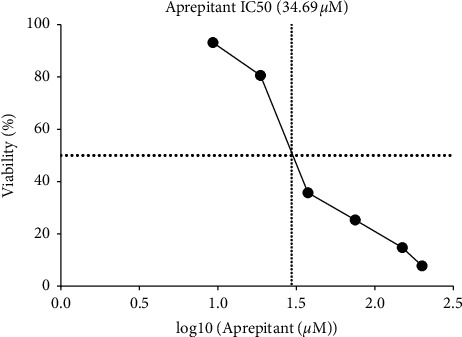
Aprepitant-induced growth inhibition and decreased viability of U87 cells. Aprepitant exerted inhibitory effects on cell viability in a concentration-dependent manner and was evaluated using resazurin assay after 24 h incubation with increasing aprepitant doses. The IC50 value for aprepitant was about 34.69 *µ*M. All results were shown as mean ± SD of three independent experiments.

In this study, the resazurin cell viability assay was used to evaluate the cytotoxic effects of aprepitant on glioblastoma cancer cells. The cell viability results with different aprepitant concentrations (0 (control), 5, 10, 25, 35, and 50 *μ*M) are shown in Figure 1. Aprepitant reduced cell viability of the U87 cell line in a dose-dependent manner. The estimated IC50 value for aprepitant in U87 cells was 34.69 *µ*M, a concentration that half of the malignant cells lost their metabolic activity in response to this NK1R antagonist. These findings were suggestive of the possible antitumor activity of aprepitant in glioblastoma-derived cells.

### 3.2. The Effect of Aprepitant on the Production of ROS in U87 Cells

**Figure 2:**
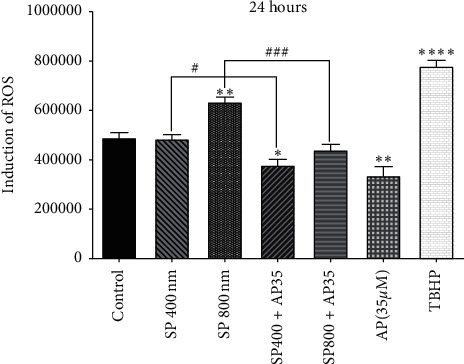
The inhibition of ROS production by aprepitant resulted in enhanced caspase-mediated apoptosis in U87 cells. Values are the mean ± SD of two independent experiments (^*∗*^*P* < 0.05 vs. control; ^*∗∗*^*P* < 0.01 vs. control; ^*∗∗∗∗*^*P* < 0.001 vs. control; ^*∗*^*P* < 0.05; ^###^*P* < 0.001).

To elucidate whether aprepitant may contribute to intracellular ROS production, we investigated the ROS production in U87 cells. As shown in Figure 2, the treatment of cells with SP (concentrations of 400 and 800 nm after 24 h) significantly increased ROS's intracellular level. Interestingly, aprepitant, with or without the pretreatment with SP, reduced intracellular ROS production. Collectively, our results suggested that aprepitant administration significantly suppressed the ROS production through inhibition of the SP/NK1R system in U87 cells.

### 3.3. The Effect of Aprepitant on Superoxide Dismutase (SOD) Gene Expression

**Figure 3:**
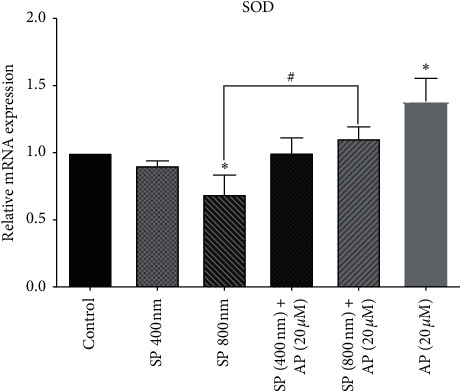
Increasing of SOD gene expression using aprepitant. After the treatment of U87 cells with aprepitant (20 *µ*M), the gene expression of the SOD enzyme notably increased as compared to untreated control cells. The levels of expression of the SOD were normalized by GAPDH mRNA levels and presented as a mean ± SD (^*∗*^*P* < 0.05 vs. control; ^#^*P* < 0.05 vs. SP800 nM).

Accumulating evidence implicates that increased ROS induced by SP stimulation is associated with decreased SOD gene expression, which plays a major role in tumor pathology [44, 45]. Accordingly, we performed qRT-PCR analysis following the administration of 20 *µ*m aprepitant in the presence or absence of different SP (400 nm and 800 nm) concentrations to measure SOD gene expression. As indicated in Figure 3, we found that SP could decrease the gene expression of SOD in the U87 cell line at the concentration of 800 nm. Moreover, to confirm that the decrease in the SOD gene expression is due to the SP/NK1R axis's stimulation, we treated the cells with aprepitant, the potent inhibitor of NK1R. We observed that the gene expression of SOD significantly increased after treatment with aprepitant 20 *µ*m, as compared to the control group and the SP 800 nm group (^*∗*^*P* < 0.05).

### 3.4. The Effect of Aprepitant on Catalase Enzyme Gene Expression

**Figure 4:**
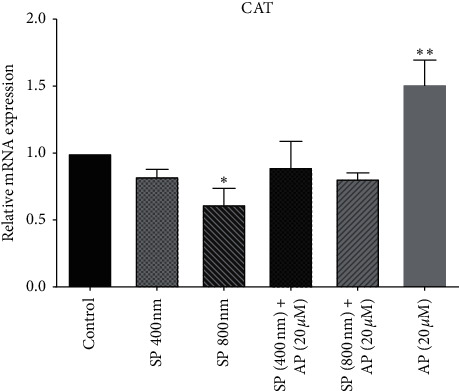
Aprepitant increases CAT gene expression. After treating U87 cells with aprepitant (20 *µ*M), the CAT enzyme gene expression notably increased compared to untreated control cells. The CAT expression levels were normalized by GAPDH mRNA levels and presented as a mean ± SD (^*∗*^*P* < 0.05 vs. control; ^*∗∗*^*P* < 0.01 vs. control).

Several lines of studies suggest that SP decreased ROS production through induced catalase gene expression, resulting in enhanced proliferation of tumor cells and decreased apoptosis [46, 47]. Accordingly, we performed qRT-PCR analysis following the administration of a 20 *µ*m aprepitant in the presence or absence of different concentrations of SP (400 nm and 800 nm) to measure CAT gene expression. As shown in Figure 4, we found that SP at the concentration of 800 nm could decrease CAT gene expression in the U87 cell line. Moreover, we observed that the gene expression of SOD significantly increased after treatment with aprepitant 20 *µ*m, as compared to the control group and the SP800 nm group (^*∗∗*^*P* < 0.01).

### 3.5. The Effect of Aprepitant on Superoxide Dismutase (SOD) Activity

**Figure 5:**
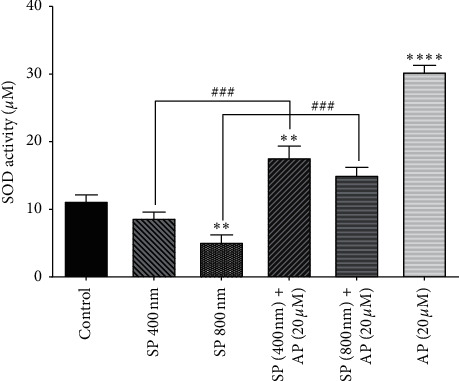
Increasing the activity of enzymes using aprepitant. After the treatment of U87 cells with aprepitant (20 *µ*M), the activity of the SOD enzyme was notably increased as compared to untreated control cells. Data were reported as the means ± SD of values derived from duplicates and are representative of three experiments (^*∗∗*^*P* < 0.01 vs. control; ^*∗∗∗∗*^*P* < 0.001 vs. control; ^###^*P* < 0.001 vs. related groups).

Superoxide dismutase is a well-known enzyme of the antioxidant system that compensates for ROS's harmful effects caused by SP [48, 49]. As evident in Figure 5, we found that culturing the cells with SP at the concentration of 800 nm caused a significant decrease in the activity of SOD. Moreover, to confirm that the decrease of SOD activity is due to the stimulation of the SP/NK1R axis in the U87 cells, we treated the cells with the antagonist of NK1R. Of note, our results showed that aprepitant (20 *µ*m), as a single agent, could increase the SOD activity in U87 cells.

### 3.6. The Effect of Aprepitant on Catalase Activity

**Figure 6:**
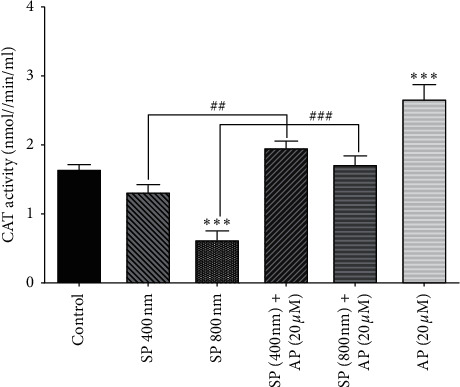
Increasing the activity of catalase using aprepitant. After treating U87 cells with aprepitant (20 *µ*M), the CAT enzyme activity was notably increased compared to untreated control cells. Data were reported as the means ± SD of values derived from duplicates and are representative of three experiments (^*∗∗∗*^*P* < 0.001 vs. control; ^##^*P* < 0.01, ^###^*P* < 0.001 vs. related groups).

Several studies indicated that catalase activity significantly decreased in many cancers through SP stimulation and increased ROS production [47, 48]. We found that SP (800 nm) could significantly reduce the CAT activity in U87 cells, and the ablation of NK1R using aprepitant (20 *µ*m) was coupled with the remarkable increase in CAT activity (Figure 6).

## 4. Discussion

In this study, we investigated the SP/NK1R system's effect on the expression and activity of catalase and superoxide dismutase in glioblastoma cancer. The results showed that substance P induced the production of reactive oxygen species (ROS) by binding to the NK1 receptor in these cells.

Glioblastoma, known as one of the most malignant brain cancers, is commonly identified and diagnosed in patients with advanced steps and final stages. In addition to its widespread invasion of the surrounding tissue and rapid progression, this type of cancer has a usual recurrence after treatment. Due to this invasive nature and lack of appropriate screening to diagnose at the early stages of the disease, glioblastoma cancer prognosis is worse than other brain cancers [50]. Considering the side effects of chemotherapy and drug resistance, especially in advanced cases, it is important to find a suitable and more effective drug to treat glioblastoma cancer [51, 52].

It is now known that the tachykinin (TK) system, which plays a crucial role in the transmission of neural messages in the central and peripheral nervous systems, may also be involved in the progression of cancers. The biological activities of SP, the most basic member of the mammalian TK peptides, are mediated through a G-protein coupled receptor (GPCR) named neurokinin-1 receptor, and it is known that the SP/NK1R system is also involved in survival, proliferation, progression, and metastasis of several human tumor cells [21, 53].

Several pharmacological agents are under assessment to block NK1R activation; among them, aprepitant is a specific, potent, and long-acting NK1R antagonist currently utilized to prevent chemotherapy-induced nausea and vomiting [54, 55]. Moreover, a remarkable number of studies focused on the antitumor properties of this drug in various cancer cell lines. In this regard, studies on glioblastoma cancer have shown that NK1R expression is increased in the brain tissue of patients, and this increase in expression is related to the size of the tumor and the extent of its invasion and spread to surrounding tissues [56]. In a study conducted by Zhu et al [44], SP's effects on ROS levels in microglial cells were evaluated. The results of this study suggested that substance P could increase ROS production in microglia. This study's findings also showed that NK1R mediated the increase in ROS production in microglial cells. Silencing this receptor's expression in these cells leads to inhibition of ROS production induced by SP [44]. In a similar vein, another recent study showed that SP increased ROS production in esophageal cancer. This increase causes oxidative stress, and as a result, it leads to tumor cell formation and cell death [45]. Thus, the use of antioxidant enzymes such as catalase and superoxide dismutase may play a major role in inhibiting ROS formation, which can prevent cancer progression by removing and inactivating reactive oxygen species [57].

In this study, we also investigated the possible effect of the SP/NK1R system on the expression of catalase and superoxide dismutase enzymes in glioblastoma cancer cells. In this regard, other studies have shown that the expression of CAT and SOD enzymes in breast cancer cells is reduced. This study showed that the decrease of these two enzymes leads to the formation of reactive oxygen species and the development of tumor cells and apoptosis [48, 58]. In this regard, in our present study, it was found that substance P can significantly reduce the expression of catalase and superoxide dismutase in U87 cells in some doses. Besides, using aprepitant (20 *μ*M) in the presence or absence of SP caused a significant increase in these two enzymes' expression in the U87 cell line.

**Figure 7:**
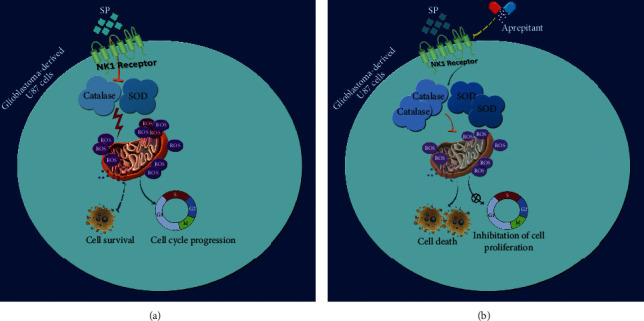
Schematic representation proposed for the plausible mechanisms through which aprepitant could prevent the effects of SP/NK1R on the antioxidant balance in glioblastoma-derived U87 cells. While the interaction between SP and NK1R in U87 cells increased the intracellular level of ROS via suppressing the enzymatic activity of catalase and SOD, blockage of NK1R using aprepitant altered the oxidative balance of the cells. As presented, aprepitant increased both the expression and the enzymatic activity of catalase and SOD, which in turn abolish the production of ROS from the mitochondria, an event that leads to the reduction in the survival and the proliferative capacity of the cells.

Also, we evaluated the potential effects of the SP/NK1R system on the activity of catalase and superoxide dismutase in the U87 cell line. Negahdar et al. examined the activity of SOD and CAT in the whole blood of 50 patients with breast cancer. The results showed that the SOD and CAT activities in BC patients were significantly lower than the control group [48]. In the study conducted by Srivastava et al., lower SOD and CAT enzyme activities were reported in patients with oral cancer from the second to the fourth stage (according to TNM) [49](Figure 7).

## 5. Conclusion

Our study results showed that SP agonists reduce catalase and superoxide dismutase activity at some doses. On the other hand, aprepitant (20 *μ*M) in the presence or absence of SP causes a significant increase in CAT and SOD enzyme activity. Taken together, it can be concluded that the inhibitors of the SP/NK1R system, such as aprepitant, might be considered as a part of therapeutic protocols in patients with glioblastoma multiforme.

## Data Availability

The data used to support the findings of this study are available from the corresponding author upon request.
