# Correction to “The Effect of SP/NK1R on the Expression and Activity of Catalase and Superoxide Dismutase in Glioblastoma Cancer Cells”

**DOI:** 10.1155/bri/9865242

**Published:** 2026-07-15

F. Korfi, H. Javid, R. Assaran Darban, and S. I. Hashemy, “The Effect of SP/NK1R on the Expression and Activity of Catalase and Superoxide Dismutase in Glioblastoma Cancer Cells,” *Biochemistry Research International* 2021, no. 1 (2021): 6620708, https://doi.org/10.1155/2021/6620708.

In the above article, several clarifications in the main text are required:

In the section “2.1. Cell Culture and Reagents,” the text

“U87 glioblastoma cancer cells were purchased from Pasteur Institute, Iran. Cells were maintained in RPMI‐1640 medium (Gibco, Grand Island, NY) supplemented with 10% fetal bovine serum (FBS) (Gibco, Grand Island, NY) and 1 mL of penicillin and streptomycin (Sigma 10,000 units penicillin and 10 mg of streptomycin/mL), incubated at 37°C with 5% CO2. SP and aprepitant were purchased from Sigma‐Aldrich Company, St. Louis, MO, USA.”

Should read:

“Materials and reagents were procured through an authorized Iranian supplier due to international sanctions and import regulations. The human glioblastoma cell line U87 MG (ATCC HTB‐14) was obtained from the Pasteur Institute Cell Bank (Tehran, Iran). Cells were maintained in RPMI‐1640 medium (Gibco, Grand Island, NY) supplemented with 10% fetal bovine serum (FBS) (Gibco, Grand Island, NY) and 1 mL of penicillin and streptomycin (Sigma 10,000 units penicillin and 10 mg of streptomycin/mL), incubated at 37°C with 5% CO2. SP and aprepitant were purchased from Sigma‐Aldrich Company, St. Louis, MO, USA. Cells were authenticated by the supplier, confirmed to be free of mycoplasma contamination, cryopreserved upon receipt, and early‐passage cells were used for all experiments.”

In the section, “2.4. Assessment of Superoxide Dismutase (SOD) and Catalase (CAT) Activity,” the text

“To measure these enzymes’ activity in the U87 cell line, commercial kits from Teb Pazhouhan Razi (TPR), Tehran, Iran, were utilized. The protocol was executed following the protocol from the manufacturer. The enzyme activity was computed as enzyme/mg protein (U/mg protein).”

This should read:

“To measure these enzymes’ activity in the U87 cell line, commercial kits from Teb Pazhouhan Razi (TPR), Tehran, Iran, were utilized. The protocol was executed following the protocol from the manufacturer. The enzyme activity was computed as enzyme/mg protein (U/mg protein). SOD activity was calculated according to the manufacturer’s protocol and expressed as U/mg protein based on inhibition percentage conversion.”

In addition, units for the *y*‐axis and legend of Figure 5 were incorrectly labelled as “μM” instead of “U/mg protein.” The correct Figure 5 is as follows:

**FIGURE 5:**
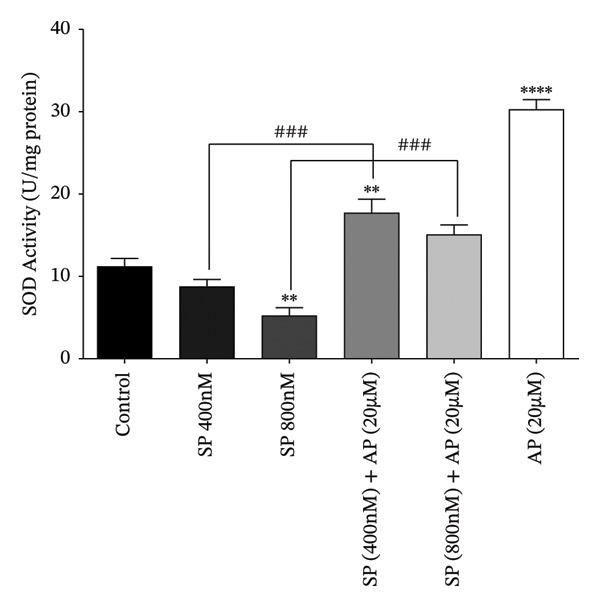
Increasing the activity of enzymes using aprepitant. After the treatment of U87 cells with aprepitant (20 U/mg protein), the activity of the SOD enzyme was notably increased as compared to untreated control cells. Data were reported as the means ± SD of values derived from duplicates and are representative of three experiments (^∗∗^ *p* < 0.01 vs. control; ^∗∗∗∗^ *p* < 0.001 vs. control; ^###^ *p* < 0.001 vs. related groups).

We apologize for these errors.

